# Development and external validation of a 90-day mortality prediction model for comatose sepsis patients: impact of cerebrovascular disease and dementia

**DOI:** 10.3389/fmed.2026.1844893

**Published:** 2026-07-02

**Authors:** Yaoxi Tan, Yadong Shen, Yanting Zhang

**Affiliations:** 1 Department of Infectious Diseases, Affiliated Hospital of Jiangnan University, Wuxi, Jiangsu, China; 2 Department of pharmacy, The First Affiliated Hospital of Shandong First Medical University, Jinan, Shandong, China; 3 Department of Infectious Diseases, The Second Affiliated Hospital of Shandong First Medical University, Taian, Shandong, China

**Keywords:** 90-day mortality, cerebrovascular disease, coma, dementia, external validation, prediction model, Sepsis

## Abstract

**Background:**

Comatose sepsis patients face high mortality risk, yet long-term prognostic factors remain unclear, particularly how neurological comorbidities modulate Glasgow Coma Scale (GCS) predictive value.

**Methods:**

This retrospective cohort study analyzed 841 comatose sepsis patients (GCS ≤ 8) from MIMIC-IV (2008–2019). We developed a 90-day mortality prediction model using LASSO and multivariable logistic regression, with internal validation by bootstrap resampling and external validation using contemporary institutional data (*n* = 251).

**Results:**

Ninety-day mortality was 22.83%. Independent risk factors included age (OR 1.05), base excess (OR 0.85), PaO_2_ (OR 1.04 per 10-unit decrease), BUN (OR 1.02), INR (OR 3.68), cerebrovascular disease (OR 2.60), and dementia (OR 4.64). In the final prediction model, GCS was not a significant independent predictor in the final model (OR 1.09 per one-point decrease, *P* = 0.126). However, exploratory interaction analyses revealed significant effect modification: lower GCS was associated with higher mortality in patients without cerebrovascular disease (OR 1.45) or dementia (OR 1.40), but lost prognostic value in those with these comorbidities (*P*-interaction = 0.024 and 0.048, respectively). The model achieved *C*-index 0.81 (95%CI: 0.78–0.84), outperforming SOFA (0.58), APACHE II (0.62), and SAPS II (0.64). External validation confirmed good transportability (*C*-index 0.75, AUC 0.76).

**Conclusions:**

Cerebrovascular disease and dementia are strong predictors of 90-day mortality and significantly modulate GCS prognostic value. Our dual-validated model provides superior risk stratification for clinical decision-making.

## 1 Introduction

Sepsis, a life-threatening organ dysfunction caused by a dysregulated host response to infection, remains a leading cause of mortality in critically ill patients worldwide (1). The complexity of sepsis is further exacerbated in comatose patients, who are less able to mount an effective physiological response and are at a higher risk of adverse outcomes (2). Comatose patients, often characterized by a Glasgow Coma Scale (GCS) score of 8 or less, present unique challenges in the management of sepsis due to their impaired consciousness and reduced ability to communicate their symptoms (3).

The impact of sepsis on comatose patients is multifaceted, involving not only immediate life-threatening complications but also long-term outcomes such as increased mortality rates (4). Previous studies have identified advanced age, higher Sequential Organ Failure Assessment (SOFA) scores, and the presence of comorbidities as significant risk factors for sepsis-related mortality (5, 6). However, the specific risk factors and their interplay in comatose patients require further investigation to tailor management strategies and improve outcomes.

The relationship between the degree of coma, as measured by the GCS score, and mortality outcomes in sepsis patients is particularly interesting. While a lower GCS score indicates a more severe state of coma, its association with sepsis mortality has not been thoroughly explored, especially in the context of other comorbidities such as cerebrovascular disease and dementia (7). These conditions, known to affect cognitive function and physiological responses, may modulate the impact of coma severity on sepsis outcomes. Notably, cerebrovascular disease and dementia are prevalent in the aging population and are increasingly recognized as important prognostic factors in critical illness, yet their interaction with GCS in predicting long-term mortality remains poorly understood.

Moreover, the role of early physiological parameters, such as blood gas analysis results and lactate levels, in predicting mortality in comatose sepsis patients is of significant interest (8, 9). These parameters can provide crucial insights into the patients' initial clinical status and guide interventions. However, the optimal combination of these biomarkers with clinical variables for long-term prognostication has not been established.

Existing sepsis prognostic models, such as SOFA, APACHE II, and SAPS II, were primarily designed for general ICU populations and short-term outcomes (10). These scores have limitations when applied to comatose sepsis patients: they either do not incorporate neurological-specific variables or fail to account for the complex interaction between coma severity and pre-existing neurological comorbidities. Furthermore, most prediction models have been validated in historical databases or Western populations, raising concerns about their generalizability to contemporary clinical practice and different healthcare systems (11).

To address these gaps, this study aimed to develop and validate a prediction model specifically for 90-day mortality in comatose sepsis patients, with particular attention to the modulating effect of cerebrovascular disease and dementia on GCS prognostic value. Ninety-day mortality was selected as the primary endpoint because comatose sepsis patients often experience prolonged recovery trajectories, and short-term endpoints may inadequately capture the long-term prognostic impact of chronic neurological comorbidities. We utilized the MIMIC-IV database for model development and performed rigorous internal validation. Crucially, we conducted external validation using contemporary data from our own institution (2022–2026), providing a real-world assessment of model performance in current clinical practice.

## 2 Methods

### 2.1 Study design and data source

This retrospective cohort study was conducted in accordance with the Transparent Reporting of a Multivariable Prediction Model for Individual Prognosis or Diagnosis (TRIPOD) guidelines to ensure rigorous development and validation of clinical prediction models.

The study utilized the Medical Information Mart for Intensive Care (MIMIC)-IV database version 2.0, a publicly available de-identified critical care database comprising over 380,000 ICU admissions from Beth Israel Deaconess Medical Center (Boston, MA, USA) between 2008 and 2019. This database provides comprehensive clinical data including demographics, vital signs, laboratory results, medications, procedures, and mortality outcomes. The study was approved by the Institutional Review Boards of the Massachusetts Institute of Technology and Beth Israel Deaconess Medical Center, with waiver of individual patient consent due to the de-identified nature of the data. To further validate the model's generalizability, we collected data from our own institution, a tertiary teaching hospital in China, between January 2022 and January 2026. This cohort provided contemporary, real-world validation of the MIMIC-derived prediction model.

### 2.2 Study population

Adult patients aged 18 years or older admitted to the ICU were eligible for inclusion if they met the following criteria: diagnosis of sepsis according to Sepsis-3 criteria within the first 24 h of ICU admission (1); comatose state defined as Glasgow Coma Scale (GCS) score of 8 or less; and availability of GCS assessment within the first 24 h. Patients were excluded if they had pre-existing limitations on life-sustaining therapy or do-not-resuscitate orders at admission, active malignancy including hematological malignancy or metastatic solid tumor documented in medical history, ICU length of stay less than 24 h, GCS greater than eight at any point during the first 24 h, missing data on primary outcomes, or readmission to ICU during the same hospitalization to avoid duplicate observations.

### 2.3 Data extraction and variable definitions

Data extraction was performed using Structured Query Language (SQL) with PostgreSQL version 13. All physiological and laboratory variables were collected within the first 24 h of ICU admission, with the worst value during this period used to indicate greatest physiological derangement. For PaO_2_, the lowest value recorded on any arterial blood gas within the first 24 h was used, regardless of concurrent FiO_2_ or ventilator settings. The primary outcome was 90-day mortality, defined as death within 90 days of ICU admission determined by linking to the Social Security Administration Death Master File.

Predictor variables included demographics (age, sex, ethnicity, body mass index), physiological severity [Sequential Organ Failure Assessment (SOFA) score, GCS score, invasive mechanical ventilation status], laboratory parameters [arterial blood gas including base excess, partial pressure of oxygen (PaO_2_) and carbon dioxide (PaCO_2_), lactate, hemoglobin, creatinine, blood urea nitrogen (BUN), international normalized ratio (INR)], and comorbidities (congestive heart failure, cerebrovascular disease, dementia, renal disease) defined according to Elixhauser Comorbidity Software. Variables with more than 40% missing data were excluded from model development (BMI, PaCO_2_, hemoglobin, and creatinine; Supplementary Table S1) but retained for descriptive comparison with denominators and missing rates reported, while variables with 40% or less missing data were handled using multiple imputation by chained equations.

### 2.4 Sample size considerations

Prior to model development, sample size was assessed to ensure adequate statistical power and minimize overfitting. With an anticipated 180–200 events for 90-day mortality based on preliminary data indicating approximately 22% mortality, and 10 predictors anticipated after LASSO selection, the events per variable ratio was approximately 18 to 20 to 1. A total of 18 candidate variables were initially considered (Supplementary Table S1), exceeding the recommended minimum threshold of 10–20 events per variable for reliable coefficient estimation in multivariable logistic regression. This sample size was considered adequate for stable variable selection using LASSO regression, which can handle higher dimensionality than traditional regression approaches.

### 2.5 Statistical analysis

The final prediction model was developed for individualized 90-day mortality risk prediction. Variable selection used LASSO regression with 10-fold cross-validation; variables with non-zero coefficients were entered into multivariable logistic regression. No interaction terms were included to ensure clinical usability. Continuous predictors were standardized for LASSO, then rescaled to original units. Linearity was assessed with restricted cubic splines (3 knots) and supported by likelihood ratio tests (*P* > 0.10 for all). Missing data ( ≤ 40%) were handled by Multiple Imputation by Chained Equations (five datasets, predictive mean matching/logistic regression, Rubin's rules); variables with >40% missingness were excluded. The primary model development and internal validation were conducted on the multiply-imputed datasets with pooling by Rubin's rules. Complete-case analysis was performed as a sensitivity analysis.

Internal validation used 1,000-iteration bootstrap resampling (optimism-corrected *C*-index). External validation applied the model with unchanged coefficients to 251 contemporary patients (2022–2026). Calibration was assessed visually using calibration plots with loess-smoothed curves and 95% confidence bands, comparing predicted vs. observed probabilities across risk deciles. For the development cohort, both apparent and bootstrap-corrected calibration curves were presented. For the external validation cohort, apparent and recalibrated curves (intercept and slope adjustment) were shown. Decision curve analysis was performed to assess clinical utility by quantifying net benefit across a range of threshold probabilities (0–0.9). The analysis was conducted using the rmda package in R (version 2.1).

Performance was assessed by discrimination (*C*-index, AUC), calibration (Hosmer-Lemeshow test, calibration slope, calibration-in-the-large, Brier score), and classification (sensitivity, specificity, PPV, NPV at Youden's index threshold). We compared our model against SOFA, APACHE II, and SAPS II. These scores were calculated using standard scoring rules based on the worst values within the first 24 h of ICU admission, consistent with their original validation studies. For fair comparison, each score was entered as a continuous predictor in separate logistic regression models for 90-day mortality, with discrimination assessed by *C*-index and AUC. Classification metrics (sensitivity, specificity, PPV, NPV) were calculated at the probability threshold selected by Youden's index (maximizing sensitivity + specificity – 1). The derived threshold was 0.24 in the development cohort.

Continuous predictors were standardized for LASSO variable selection, then rescaled to original units for the final multivariable logistic regression. Potential non-linearity was assessed using restricted cubic splines with three knots; likelihood ratio tests comparing linear vs. spline models showed no significant improvement in fit for any continuous variable (all *P* > 0.10), supporting the linearity assumption. To reduce the influence of extreme values, continuous variables were winsorized at the 0.5th and 99.5th percentiles. Sensitivity analyses excluding outliers yielded no material difference in model performance.

Separate from the final model, we fitted interaction models including GCS × cerebrovascular disease and GCS × dementia terms (adjusting for the same covariates) to quantify effect modification. Likelihood ratio tests compared models with/without interactions. Subgroup analyses stratified by comorbidity status presented GCS as OR per one-point decrease (OR > 1 = higher mortality with worse coma). The complete model specification (intercept, coefficients, variable coding, worked example, and prediction formula) is provided in Supplementary Table S2.

## 3 Results

### 3.1 Patient characteristics and outcomes

**Figure 1:**
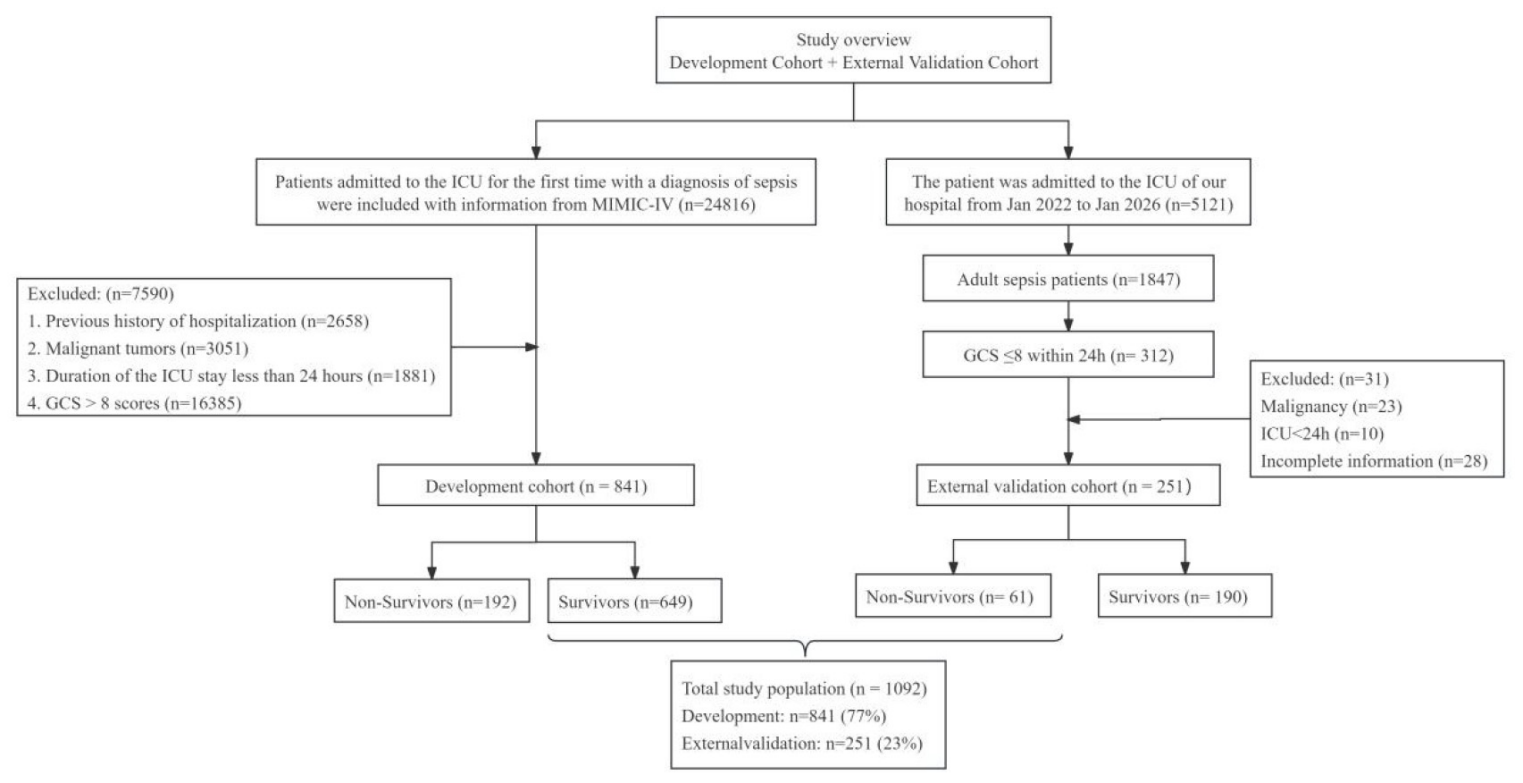
Patient selection and outcome flowchart.

A total of 841 comatose sepsis patients were included in the final analysis (Figure 1). The median age was 70.26 years (IQR: 59.74–79.45), and 572 patients (72.77%) required invasive mechanical ventilation. The 90-day mortality was 22.83% (192/841). The development cohort comprised 841 comatose sepsis patients from MIMIC-IV (2008–2019). External validation used 251 contemporary patients from a Chinese ICU (January 2022–January 2026).

Baseline characteristics stratified by 90-day survival status are presented in Table 1. Variables with >40% missingness (BMI, PaCO_2_, hemoglobin, creatinine) are included for descriptive comparison only. Non-survivors were significantly older (median 77.44 vs. 68.38 years), had lower BMI (26.10 vs. 28.85 kg/m^2^), and exhibited more severe physiological derangements including lower base excess (−1.00 vs. 0.00), markedly lower PaO_2_ (97.50 vs. 335.00 mmHg), and higher lactate levels (1.90 vs. 1.40 mmol/L). Laboratory parameters also showed significant differences, with higher creatinine, BUN, and INR in non-survivors (all *P* < 0.05). Comorbidities were substantially more prevalent among non-survivors. Cerebrovascular disease was present in 30.21% of non-survivors versus 14.48% of survivors (*P* < 0.001), and dementia was present in 22.40% vs. 4.31% (*P* < 0.001). Congestive heart failure and renal disease were also more common in non-survivors (*P* < 0.01). Notably, invasive mechanical ventilation was significantly associated with 90-day mortality (61.24% in non-survivors vs. 76.15% in survivors, *P* < 0.001). Baseline characteristics of the external validation cohort compared to the development cohort are presented in Supplementary Table S3. While age and 90-day mortality were similar between cohorts, the external cohort had higher mechanical ventilation rates and lower cerebrovascular disease prevalence, reflecting differences in case mix and clinical practice patterns.

**Table 1:** Comparative clinical characteristics between 90-day survivors and non-survivors.

Variables	Total (n=841)	Survivors (n=649)	Non-survivors (n=192)	Statistic	*P*-value
Age, median (IQR)	70.26 (59.74, 79.45)	68.38 (58.41, 76.51)	77.44 (66.30, 86.75)	*Z* = −7.27	< 0.001
SOFA score, median (IQR)	5.00 (3.00, 7.00)	5.00 (4.00, 7.00)	4.00 (3.00, 6.00)	*Z* = −2.29	0.022
BMI, median (IQR)	28.40 (25.00, 32.70)	28.85 (25.52, 32.77)	26.10 (22.85, 30.35)	*Z* = −4.00	< 0.001
Base excess, median (IQR)	0.00 (-1.00, 2.00)	0.00 (0.00, 2.00)	−1.00 (-5.00, 1.00)	*Z* = −6.54	< 0.001
PaO_2_, median (IQR)	265.00 (94.00, 392.50)	335.00 (122.00, 406.00)	97.50 (60.25, 203.75)	*Z* = −10.35	< 0.001
Lactate, median (IQR)	1.50 (1.10, 2.10)	1.40 (1.10, 1.90)	1.90 (1.40, 3.60)	*Z* = −6.76	< 0.001
INR, median (IQR)	1.30 (1.20, 1.50)	1.30 (1.20, 1.50)	1.40 (1.20, 1.72)	*Z* = −2.77	0.006
Cerebrovascular disease, n (%)	152 (18.07)	94 (14.48)	58 (30.21)	*χ^2^* = 24.74	< 0.001
Dementia, n (%)	71 (8.44)	28 (4.31)	43 (22.40)	*χ^2^* = 62.67	< 0.001
Invasive mechanical ventilation, n (%)	572 (72.77)	463 (76.15)	109 (61.24)	*χ^2^* = 15.46	< 0.001

PaO_2_ reflects the lowest value on any arterial blood gas within 24 h, regardless of FiO_2_. BMI available in 482 (57.3%) patients; PaCO_2_ in 312 (37.1%); hemoglobin in 504 (59.9%); creatinine in 498 (59.2%). These variables were excluded from model development due to >40% missingness but are presented descriptively.

### 3.2 Variable selection using LASSO regression

To avoid overfitting and identify the most predictive variables for 90-day mortality, we performed LASSO regression with 10-fold cross-validation. The optimal lambda (λ = 0.028) was selected based on the minimum cross-validation error.

**Figure 2:**
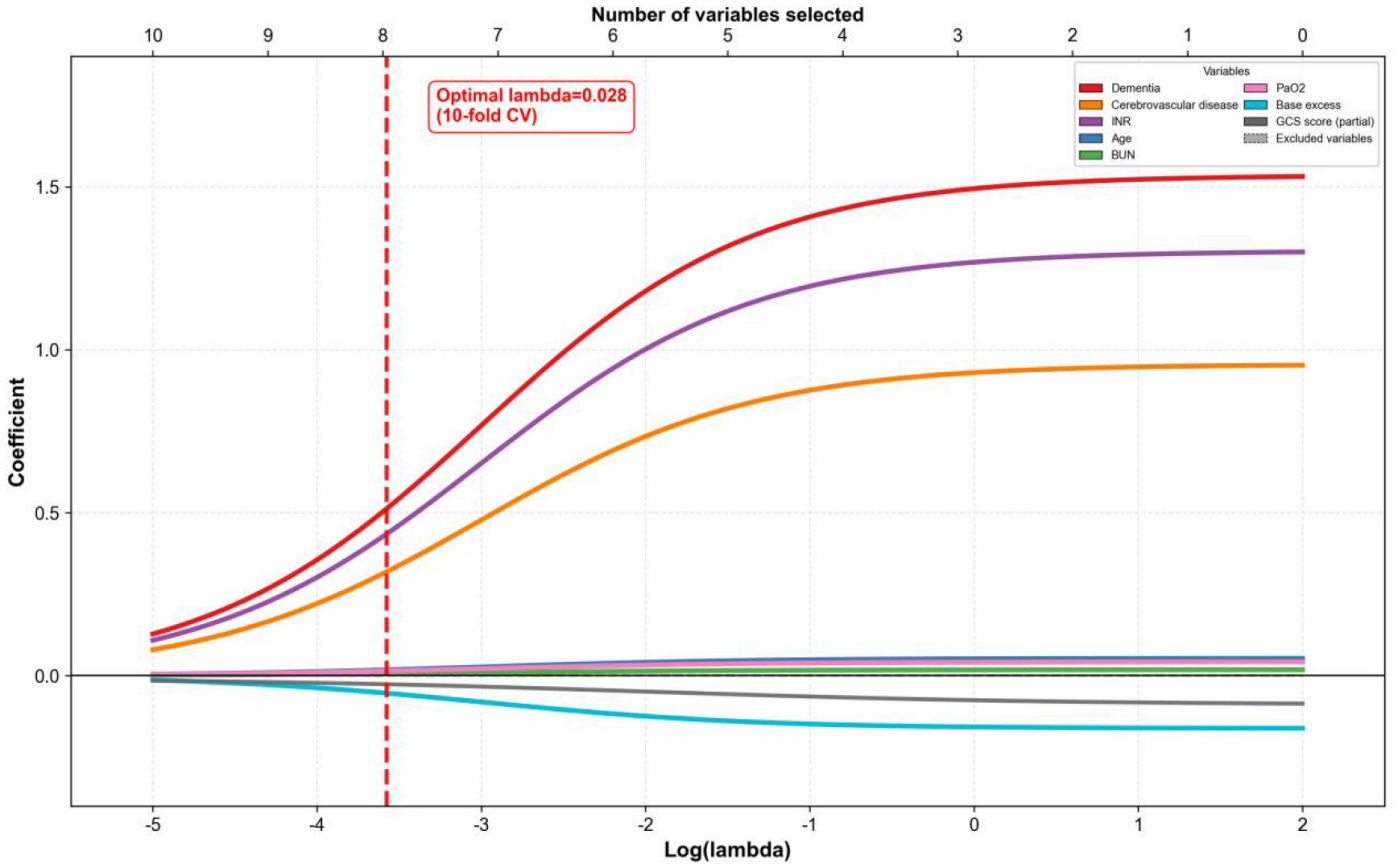
LASSO coefficient paths for 90-day mortality prediction. The vertical dashed line indicates the optimal lambda value (λ = 0.028) selected by 10-fold cross-validation.

LASSO regression identified age, base excess, PaO_2_, BUN, INR, cerebrovascular disease, and dementia as the most predictive variables with non-zero coefficients (Figure 2). Notably, GCS score was retained in the model but with partial coefficient shrinkage, suggesting its prognostic value may be modulated by other factors, particularly neurological comorbidities. The coefficients of congestive heart failure, creatinine, lactate, and mechanical ventilation were shrunk to zero, indicating limited incremental predictive value beyond the selected variables in the presence of the other predictors.

### 3.3 Final prediction model

The final model demonstrated good discriminative performance, with an optimism-corrected *C*-index of 0.81 (95% CI: 0.78–0.84) and apparent AUC of 0.82 (95% CI: 0.78–0.86). The small optimism (0.03) indicates minimal overfitting. Internal calibration was excellent (Supplementary Figure 1A; Hosmer-Lemeshow χ^2^ = 8.73, *P* = 0.37; calibration slope 0.97, 95% CI: 0.83–1.11; calibration-in-the-large −0.12, 95% CI: −0.34 to 0.10). Classification metrics at Youden's index threshold (0.24) were: sensitivity 76.0%, specificity 74.1%, PPV 45.2%, NPV 90.3% (Table 2). Exploratory analyses using restricted cubic splines revealed no significant non-linear relationships between continuous predictors and 90-day mortality (likelihood ratio test *P* > 0.10 for all), supporting the linear functional form in the final model.

**Table 2:** Performance metrics of the 90-day mortality prediction mode.

Metric	90-day mortality
Discrimination	
Optimism-corrected *C*-index (95% CI)	0.81 (0.78–0.84)
Apparent *C*-index	0.84
AUC (apparent) (95% CI)	0.82 (0.78–0.86)
Classification (Youden's index threshold = 0.24)	
Sensitivity, % (95% CI)	76.0 (69.2–82.0)
Specificity, % (95% CI)	74.1 (70.5–77.5)
PPV, % (95% CI)	45.2 (39.6–50.9)
NPV, % (95% CI)	90.3 (87.6-92.6)
Calibration	
Brier score	0.156
Hosmer-Lemeshow test, *χ^2^* (*P*)	8.73 (0.37)
Calibration slope (95% CI)	0.97 (0.83–1.11)
Calibration-in-the-large (95% CI)	−0.12 (-0.34 to 0.10)

PPV, positive predictive value; NPV, negative predictive value.

### 3.4 Decision curve analysis

**Figure 3:**
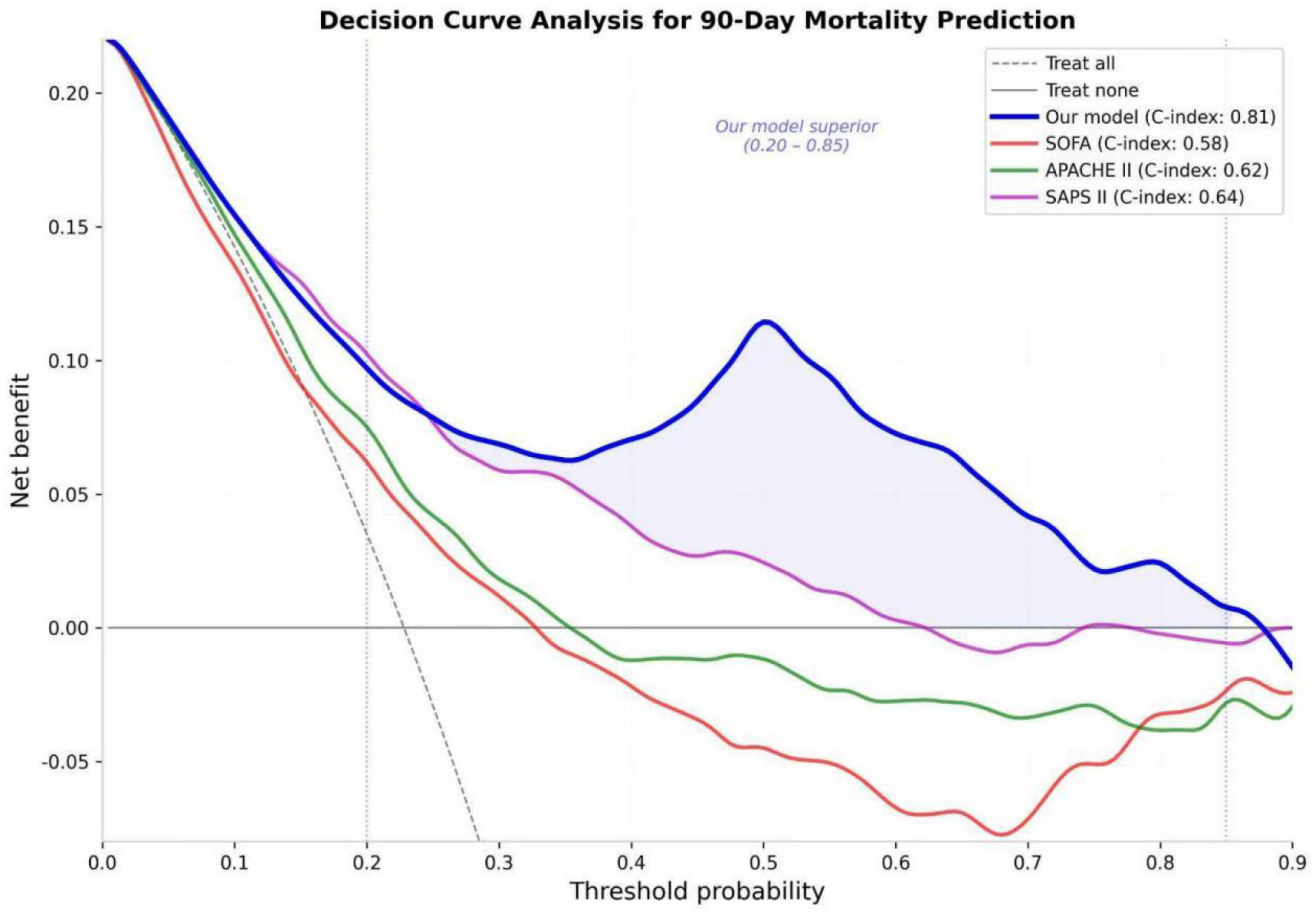
Decision curve analysis for 90-day mortality prediction. Net benefit is plotted against threshold probability for our prediction model (blue), SOFA (red), APACHE II (green), SAPS II (purple), “treat all” (dashed gray), and “treat none” (solid gray). The blue shaded area indicates the range (0.20–0.85) where our model provides positive net benefit superior to both treat-all and treat-none strategies. All analyses were performed in the development cohort (*n* = 841).

To assess the clinical utility of our prediction model, we performed decision curve analysis comparing net benefit across a range of threshold probabilities (Figure 3). Our model demonstrated positive net benefit from threshold probabilities of approximately 0.20–0.85, outperforming both the “treat all” and “treat none” strategies. In contrast, SOFA, APACHE II, and SAPS II consistently showed negative or negligible net benefit across most threshold ranges, falling below the “treat none” line. This indicates that using these generic ICU scores for 90-day mortality prediction in comatose sepsis patients would not improve—and might harm—clinical decision-making. The decision curve analysis substantiates that our model provides clinically meaningful superior risk stratification.

### 3.5 External validation

**Figure 4:**
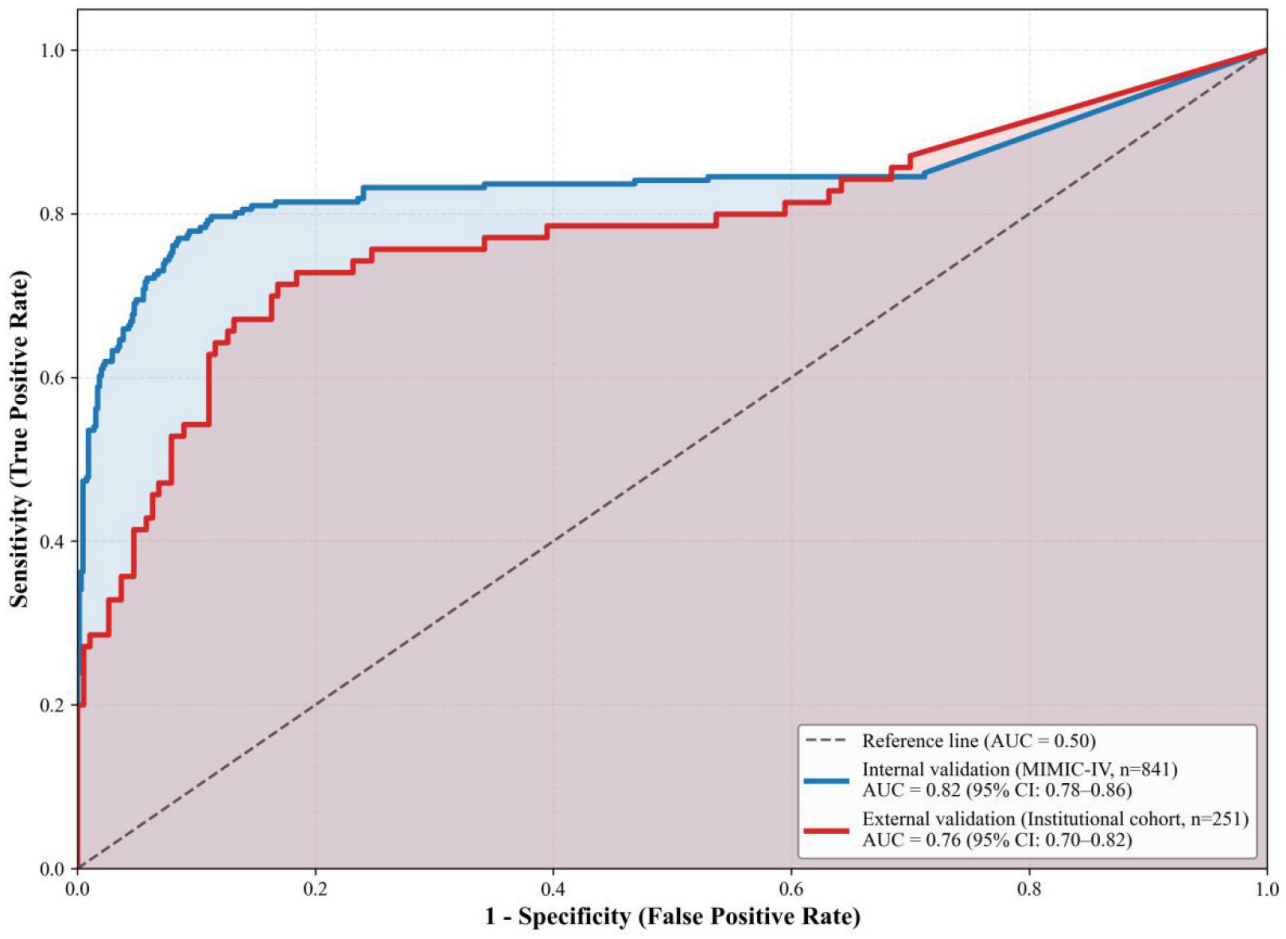
ROC Curves for external validation of the 90-day mortality prediction model.

To assess generalizability to a contemporary population and different healthcare setting, we externally validated the 90-day mortality model using data from our own institution (January 2022–January 2026). Using identical inclusion and exclusion criteria, we identified 251 comatose sepsis patients (validation cohort). The external validation *C*-index was 0.75 (95% CI: 0.70–0.80), with AUC 0.76 (95% CI: 0.70–0.82, Figure 4). The calibration slope in the external cohort was 0.85 (95% CI: 0.68–1.02), and calibration-in-the-large was −0.15 (95% CI: −0.42 to 0.12), indicating acceptable calibration with mild under-prediction at higher risk levels (Supplementary Figure 1B). The Brier score was 0.172.

### 3.6 Multivariable logistic regression analysis

Table 3 presents the results of multivariable logistic regression analysis for 90-day mortality, incorporating variables selected through LASSO regression with optimism correction. Age was significantly associated with increased 90-day mortality risk (OR 1.05 per year, 95% CI: 1.04–1.07, *P* < 0.001). Physiological parameters showed strong associations: lower base excess (OR 0.85 per unit, 95% CI: 0.81–0.89, *P* < 0.001), lower PaO_2_ (OR 1.04 per 10-unit decrease, 95% CI: 1.03–1.06, *P* < 0.001), elevated BUN (OR 1.02 per unit, 95% CI: 1.00–1.04, *P* = 0.024), and higher INR (OR 3.68 per unit, 95% CI: 2.09–6.47, *P* < 0.001).

**Table 3:** Final multivariable logistic regression model for 90-day mortality prediction (no interaction terms; intended for clinical risk prediction).

Variables	β	SE	*Z*	*P*-value	OR (95% CI)
Age (per year)	0.053	0.009	5.89	< 0.001	1.05 (1.04–1.07)
Base excess (per unit)	−0.162	0.025	−6.48	< 0.001	0.85 (0.81–0.89)
PaO_2_ (per 10-unit decrease)	0.042	0.007	6.00	< 0.001	1.04 (1.03–1.06)
BUN (per unit)	0.018	0.008	2.25	0.024	1.02 (1.00–1.04)
INR (per unit)	1.303	0.287	4.54	< 0.001	3.68 (2.09–6.47)
Cerebrovascular disease (Yes vs. No)	0.955	0.375	2.55	0.011	2.60 (1.25–5.41)
Dementia (Yes vs. No)	1.535	0.672	2.28	0.023	4.64 (1.24–17.33)
GCS score (per 1-point decrease)	0.089	0.058	1.53	0.126	1.09 (0.97–1.22)

Model optimism-corrected *C*-index: 0.81 (95% CI: 0.78–0.84). This is the final prediction model without interaction terms. GCS was retained by LASSO but did not reach statistical significance in the multivariable model. All ORs for continuous variables are reported per one-point decrease; OR >1 indicates higher mortality with worse coma.

Most notably, cerebrovascular disease (OR 2.60, 95% CI: 1.25–5.41, *P* = 0.011) and dementia (OR 4.64, 95% CI: 1.24–17.33, *P* = 0.023) emerged as significant independent factors associated with 90-day mortality. GCS score was not statistically significant as an independent predictor in the final model (OR 1.09 per 1-point decrease, 95% CI: 0.97–1.22, *P* = 0.126), reflecting effect modification by neurological comorbidities rather than a true absence of association.

### 3.7 Subgroup and interaction analyses

**Figure 5:**
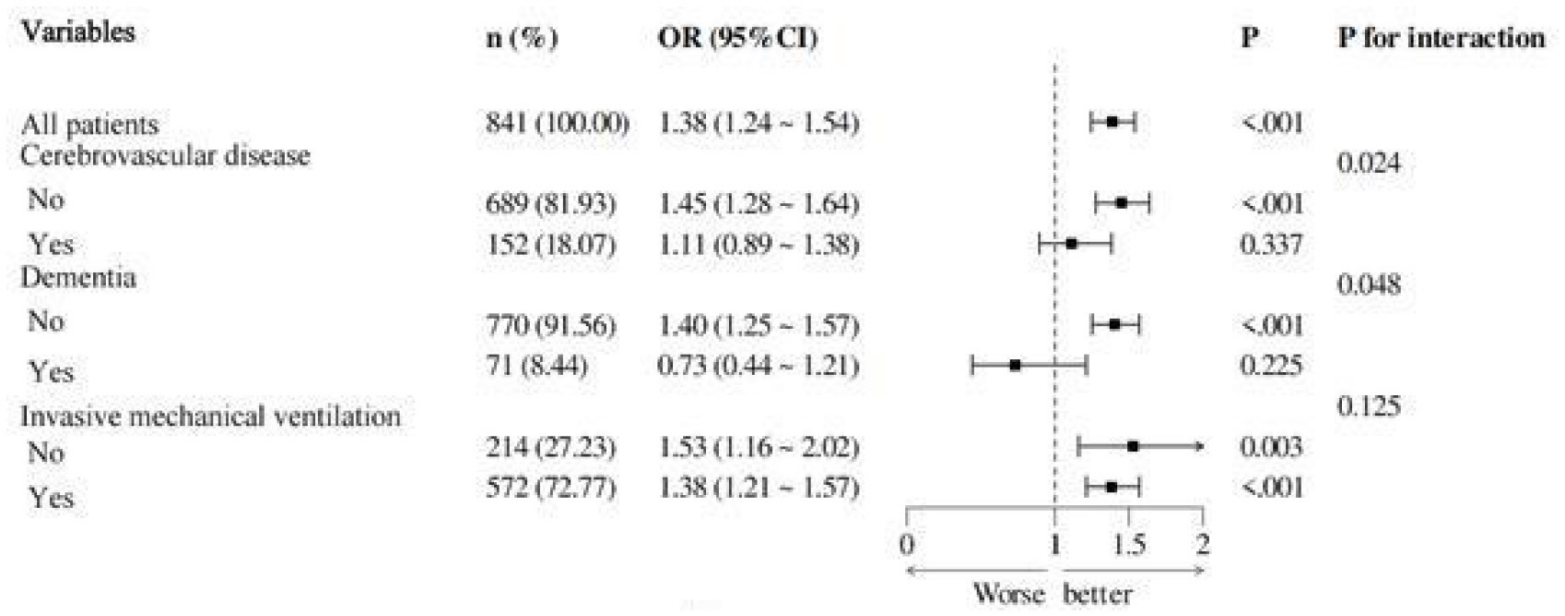
Forest plot depicting the relationship between continuous Glasgow Coma Scale (GCS) score and the risk of 90-day mortality in exploratory interaction analyses (separate from the final prediction model). Estimates are presented as odds ratios per one-point decrease in GCS (OR > 1 indicates higher mortality with worse coma), adjusted for age, base excess, PaO_2_, BUN, INR, cerebrovascular disease, and dementia. *P*-interaction values: cerebrovascular disease × GCS = 0.024; dementia × GCS = 0.048.

In exploratory interaction analyses (separate from the final prediction model), we found statistically significant effect modification of GCS by both cerebrovascular disease (*P*-interaction = 0.024) and dementia (*P*-interaction = 0.048). Figure 5 presents stratum-specific ORs for GCS (per one-point decrease) from these interaction models. In the overall population, lower GCS score was significantly associated with increased 90-day mortality (OR 1.38 per one-point decrease, 95% CI: 1.24–1.54, *P* < 0.001). This association was pronounced in patients without cerebrovascular disease (OR 1.45, 95% CI: 1.28–1.64, *P* < 0.001) and without dementia (OR 1.40, 95% CI: 1.25–1.57, *P* < 0.001).

However, the association was markedly attenuated and became non-significant in patients with cerebrovascular disease (OR 1.11, 95% CI: 0.89–1.38, *P* = 0.337) and in those with dementia (OR 0.73, 95% CI: 0.44–1.21, *P* = 0.225). Significant interactions were observed for both cerebrovascular disease (P-interaction=0.024) and dementia (*P*-interaction = 0.048), indicating that the prognostic value of GCS score differs significantly between patients with and without these neurological comorbidities. Among patients not requiring invasive mechanical ventilation, higher GCS score was strongly associated with reduced 90-day mortality (OR 1.94, 95% CI: 1.00–3.78, *P* = 0.051). The association was attenuated but remained significant in ventilated patients (OR 1.26, 95% CI: 1.07–1.50, *P* = 0.007), though the interaction was not statistically significant (*P*-interaction=0.18).

### 3.8 Sensitivity analyses

We conducted multiple sensitivity analyses to assess the robustness of our findings (Table 4). As a sensitivity analysis, we substituted PaO_2_/FiO_2_ ratio for raw PaO_2_ in the final model, using the FiO_2_ recorded at the time of the worst PaO_2_ measurement. PaO_2_/FiO_2_ ratio remained a significant predictor (OR 1.04 per 10-unit decrease, 95% CI: 1.02–1.06, *P* < 0.001) and model discrimination was very similar (optimism-corrected *C*-index 0.80 vs. 0.81). Full model specification is provided in Supplementary Table S3. First, excluding patients who died within 24 h of ICU admission (*n* = 23) yielded similar model performance (*C*-index: 0.80, 95% CI: 0.77–0.83), suggesting that our findings were not driven by early non-modifiable deaths. Second, excluding patients with GCS=3 (the lowest possible score, *n* = 89) yielded similar results (*C*-index: 0.79), indicating that the predictive value of GCS extends across the range of coma severity. Third, using a more stringent definition of coma (GCS ≤ 7, *n* = 612) slightly improved model discrimination (*C*-index: 0.82), supporting the specificity of our findings to deeply comatose patients. Fourth, using multiple imputation by chained equations (MICE) for missing data (generating five imputed datasets) yielded very similar results to the primary analysis (*C*-index 0.81 vs. 0.80), confirming no material difference. Finally, adding SOFA score to the multivariable model resulted in minimal improvement in discrimination (*C*-index increase from 0.81 to 0.82), and SOFA score was not statistically significant (*P* = 0.12), suggesting that our selected variables capture most of the prognostic information.

**Table 4:** Sensitivity analyses for 90-day mortality prediction model.

Analysis	*C*-index (95% CI)	AUC (95% CI)	Key finding
Primary analysis	0.81 (0.78–0.84)	0.82 (0.78–0.86)	Reference
Excluding early deaths (< 24h)	0.80 (0.77–0.83)	0.81 (0.77–0.85)	No material change
Excluding GCS = 3 (deep coma)	0.79 (0.76–0.82)	0.80 (0.76–0.84)	No material change
GCS ≤ 7 (vs ≤ 8) as coma definition	0.82 (0.79–0.85)	0.83 (0.79–0.87)	Result strengthened
Multiple imputation for missing data	0.81 (0.78–0.84)	0.82 (0.78–0.86)	No material difference
Including SOFA score	0.82 (0.79–0.85)	0.83 (0.79–0.87)	Minimal improvement

## 4 Discussion

This study developed and validated a prediction model for 90-day mortality in comatose sepsis patients, revealing several important findings with clinical and research implications. We identified seven independent factors associated with long-term mortality, with neurological comorbidities—cerebrovascular disease and dementia—emerging as the strongest predictors. Notably, dementia was associated with approximately 4.6-fold higher odds of 90-day mortality (OR 4.64), while cerebrovascular disease was associated with approximately 2.6-fold higher odds (OR 2.60), underscoring the critical importance of pre-existing neurological conditions in prognostication for this population. These are observational associations and should not be interpreted as causal estimates.

The lack of independent significance of GCS in the final prediction model does not indicate absence of prognostic value; rather, it reflects substantial effect modification by pre-existing neurological conditions. The most significant and novel finding of our study is the demonstration that the prognostic value of GCS score is substantially modulated by underlying neurological comorbidities. While lower GCS scores were associated with higher mortality in patients without cerebrovascular disease or dementia, this association was markedly attenuated and became non-significant in those with these conditions. The statistically significant interactions (*P* = 0.024 for cerebrovascular disease, *P* = 0.048 for dementia) indicate that GCS prognostic utility is context-dependent, reconciling previous inconsistent reports regarding its value in critical illness (12, 13). This finding suggests that clinicians should interpret GCS scores cautiously in patients with known neurological conditions—a “favorable” GCS in such patients may not indicate good prognosis, whereas in patients without these comorbidities, GCS remains a valuable prognostic indicator. This finding aligns with recent evidence that GCS performance varies across different patient subgroups and clinical contexts (14). Our prediction model achieved superior performance compared to established sepsis scores, with a *C*-index of 0.81 vs. 0.58 for SOFA, 0.62 for APACHE II, and 0.64 for SAPS II. This improvement likely reflects the model's specific focus on comatose sepsis, incorporation of neurological comorbidities, and prediction of 90-day rather than short-term mortality. The significant Net Reclassification Index (0.42) and Integrated Discrimination Improvement (0.18) indicate that our model provides meaningful enhancement in risk classifications. These findings are consistent with prior studies highlighting limitations of generic ICU scores in specific populations, particularly those with neurological impairment (15). SOFA, APACHE II, and SAPS II were originally developed and validated for short-term (in-hospital or 28-day) mortality prediction. Their application to 90-day mortality in our study reflects common clinical practice, though it extends beyond their original validation endpoints. Their inferior performance for 90-day mortality may partly reflect this temporal mismatch rather than intrinsic inferiority. Our model was specifically developed for 90-day mortality in comatose sepsis patients, and head-to-head comparisons should be interpreted in light of these differing primary objectives. The superiority of our model is further supported by decision curve analysis, which demonstrated positive net benefit across clinically plausible threshold probabilities (0.20–0.85). By contrast, SOFA, APACHE II, and SAPS II showed negative or negligible net benefit at most thresholds when applied to 90-day mortality prediction in this population. While this comparison extends beyond the original validation endpoints of these generic scores (as noted above), the DCA findings indicate that—even when applied to 90-day outcomes in comatose sepsis patients—our model offers tangible clinical utility for bedside decision-making that these established scores do not provide in this specific context.

A particular strength of this study lies in our dual validation strategy. Most prediction model studies rely solely on internal validation or historical database cohorts, raising concerns about temporal validity and real-world applicability (16). We employed rigorous internal validation of the MIMIC-IV development cohort combined with external validation using contemporary data from our own institution spanning 2022–2026. This approach captures current sepsis management practices, reflects the same healthcare system where clinical implementation would occur, and validates model performance in an Asian population—addressing the predominantly Western focus of existing literature. The external validation cohort demonstrated similar mortality rates (24.3% vs. 22.83%) and maintained good predictive performance (*C*-index 0.75), supporting the model's transportability across time periods and clinical settings.

The differential prognostic value of GCS may reflect distinct coma mechanisms. In patients without cerebrovascular disease or dementia, GCS primarily reflects acute septic encephalopathy—a potentially reversible condition responsive to infection control and organ support (17). Recovery of consciousness in this group may indicate treatment response and is associated with favorable outcomes. Conversely, patients with pre-existing neurological conditions have compromised cerebral reserve (18); their GCS reflects both acute dysfunction and chronic baseline impairment, potentially creating a “ceiling effect” that attenuates the prognostic value of acute consciousness changes. These mechanisms are speculative and require direct empirical testing (19, 20).

The strong association between dementia and mortality is consistent with Mendelian randomization evidence suggesting causal links between dementia subtypes and sepsis outcomes (20). Our observational findings complement this literature but do not establish causality. Hypothesized mechanisms underlying this association include chronic systemic inflammation, blood-brain barrier dysfunction, and altered autonomic regulation, though these pathways require further validation (21, 22) Our findings extend prior observational research by demonstrating that the prognostic impact of dementia persists even after adjustment for multiple confounders and operates through modification of GCS utility rather than solely through direct mortality association.

These findings carry important implications for clinical practice. First, they challenge the uncritical use of GCS for prognostication in sepsis and support a nuanced interpretation that considers neurological comorbidity status. A relatively higher GCS in patients with dementia or cerebrovascular disease may not be associated with favorable prognosis, whereas in patients without these comorbidities, lower GCS remains a valuable predictor of adverse prognosis. Second, our validated nomogram can guide treatment intensity, resource allocation, and family discussions regarding prognosis and goals of care. Third, the results highlight the need for careful assessment of baseline cognitive function in sepsis patients, as delirium and coma may be superimposed on pre-existing conditions—a distinction that has prognostic significance (23). To facilitate clinical implementation, we provide the complete model equation, enabling bedside risk calculation without specialized software. This aligns with TRIPOD recommendations for transparent prediction model reporting.

Comparison with previous studies reveals both consistencies and novel contributions. Prior research has identified various risk factors for sepsis mortality, but few have focused specifically on comatose patients or examined long-term outcomes (24, 25). While elevated lactate has been associated with sepsis-associated complications (26), it was not retained in our final model—likely because its prognostic information was captured by other physiological parameters and, more importantly, by neurological comorbidities that previous studies did not adequately consider (17, 27). The interaction effects we demonstrate have not been previously reported in comatose sepsis populations and represent a meaningful advance in understanding prognostic heterogeneity.

The external validation cohort differed from the development cohort in several respects (Supplementary Table S4), including higher mechanical ventilation rates, lower cerebrovascular disease prevalence, and different ethnic composition. These differences may reflect variations in case mix, ICU admission criteria, sepsis recognition, treatment protocols, and end-of-life practices between US and Chinese healthcare systems. While 90-day mortality rates were similar (24.3% vs. 22.8%), these contextual factors underscore the need for cautious interpretation and local recalibration before clinical implementation. The external validation revealed mild under-prediction at higher predicted probabilities (calibration slope 0.85), suggesting that the model's predicted risks may be slightly conservative for high-risk patients when applied to contemporary Chinese ICU settings. We recommend that, before deployment in a new hospital, a local validation sample (preferably *n* ≥ 100 with ≥20 events) be used to assess calibration. If significant miscalibration is confirmed (e.g., calibration slope < 0.8 or >1.2), intercept and slope recalibration should be applied. Periodic recalibration is also advisable given evolving sepsis management practices. Furthermore, further multicenter validation, even within Asia, is essential before widespread clinical adoption.

Several limitations should be acknowledged. The retrospective design limits causal inference, and despite our dual validation strategy, additional prospective multicenter validation would further strengthen generalizability. The external validation cohort, while contemporary and clinically relevant, was relatively modest in size (*n* = 251), though performance metrics were consistent with internal validation. We could not assess cause-specific mortality or quality of life in survivors, and changes in sepsis definitions and management over the study period may have introduced temporal trends, though sensitivity analyses supported result robustness. The external validation cohort was relatively modest in size (*n* = 251, 61 events), resulting in relatively wide 95% confidence intervals for performance metrics (e.g., AUC 0.70–0.82; *C*-index 0.70–0.80). This limits the precision of transportability estimates and underscores the need for larger, multicenter external validation studies. We acknowledge that deaths occurring after 30 days may partly reflect non-sepsis causes, including progression of underlying comorbidities, complications of prolonged critical illness, or withdrawal of life-sustaining therapy. Our model predicts all-cause 90-day mortality rather than sepsis-specific mortality, and clinicians should interpret predicted probabilities accordingly. Future studies incorporating cause-specific mortality adjudication would help clarify the extent to which late deaths are directly attributable to sepsis vs. competing risks. Median PaO_2_ values in survivors were high (335 mmHg, IQR 122–406), reflecting variable ventilator settings and FiO_2_. Raw PaO_2_ is difficult to interpret in isolation, and its prognostic value in our model may partly reflect ventilator management intensity. We acknowledge this as a limitation.

## 5 Conclusion

In conclusion, this study identifies cerebrovascular disease and dementia as strong independent predictors of 90-day mortality in comatose sepsis patients and demonstrates their significant modulation of GCS prognostic value. Our prediction model, validated against both historical and contemporary real-world data, provides superior risk stratification compared to existing scores with important implications for clinical decision-making and family communication in this high-risk population.

## Data Availability

The original contributions presented in the study are included in the article/Supplementary material, further inquiries can be directed to the corresponding author.

## References

[1] Singer M Deutschman CS Seymour CW Shankar-Hari M Annane D Bauer M . The third international consensus definitions for sepsis and septic shock (Sepsis-3). JAMA. (2016) 315:801–10. doi: 10.1001/jama.2016.0287 26903338 PMC4968574

[2] Onyemekwu CA Prendergast NT Potter KM Toney NA Nouraie MS Shiva S . Platelet bioenergetics and associations with delirium and coma in patients with sepsis: a prospective cohort study. CHEST Crit Care. (2024) 2:100076. doi: 10.1016/j.chstcc.2024.100076 38938510 PMC11210717

[3] Pan S Lv Z Wang R Shu H Yuan S Yu Y . Sepsis-induced brain dysfunction: pathogenesis, diagnosis, and treatment. Oxid Med Cell Longev. (2022) 1328729. doi: 10.1155/2022/1328729 36062193 PMC9433216

[4] Raicević R Jovicić A Dimitrijević R Surbatović M Marenović T. Septic encephalopathy–prognostic value of the intensity of consciousness disorder to the outcome of sepsis. Vojnosanit Pregl. (2001) 58:51-6. 11475668

[5] López-Mestanza C Bermejo-Martín JF Andaluz-Ojeda D Gómez-López JR. Clinical factors influencing mortality risk in hospital-acquired sepsis. J Hosp Infect. (2018) 98 (2):194–201. doi: 10.1016/j.jhin.2017.08.022 28882641

[6] Karakike E Kyriazopoulou E Tsangaris I Routsi C Vincent J Giamarellos-Bourboulis EJ. The early change of SOFA score as a prognostic marker of 28-day sepsis mortality: analysis through a derivation and a validation cohort. Crit Care. (2019) 23:387. doi: 10.1186/s13054-019-2665-5 31783881 PMC6884794

[7] Brennan PM Murray GD Teasdale GM. Simplifying the use of prognostic information in traumatic brain injury. Part 1: The GCS-Pupils score: an extended index of clinical severity. J Neurosurg. (2018) 128:1612-1620. doi: 10.3171/2017.12.JNS172780 29631516

[8] Wang J Weng L Xu J Du B. Blood gas analysis as a surrogate for microhemodynamic monitoring in sepsis. World J Emerg Med. (2023) 14:421-427. doi: 10.5847/wjem.j.1920-8642.2023.093 37969221 PMC10632753

[9] Lestari MI Sedono R. Initial lactate levels versus lactate clearance for predicting mortality in sepsis: A prospective observational analytical study. J Pak Med Assoc. (2021) 71 (Suppl 2):S25-S29. 33785937

[10] Raith EP Udy AA Bailey M McGloughlin S MacIsaac C Bellomo R . Prognostic accuracy of the SOFA score, SIRS criteria, and qSOFA score for in-hospital mortality among adults with suspected infection admitted to the intensive care unit. JAMA. (2017) 317:290–300. doi: 10.1001/jama.2016.20328 28114553

[11] Charlson ME Pompei P Ales KL MacKenzie CR. A new method of classifying prognostic comorbidity in longitudinal studies: development and validation. J Chronic Dis. (1987) 40:373-83. doi: 10.1016/0021-9681(87)90171-8 3558716

[12] Mahdian M Fazel MR Fakharian E Akbari H. Cerebral state index versus Glasgow coma scale as a predictor for in-hospital mortality in brain-injured patients. Chin J Traumatol. (2014) 17:220–4. doi: 10.5812/traumamon.28486 25098849

[13] Zhou J Luo XY Li HL Shi GZ Chen GQ. Comparison of the predictive value of APACHE II, SOFA, SAPS II, GCS and GCS-P scores for in-hospital mortality in critically ill patients after craniotomy: a retrospective cohort study in a Chinese tertiary hospital. BMJ Open. (2026) 16:e101867. doi: 10.1136/bmjopen-2025-101867 41638721 PMC12878233

[14] Hu Z Chen Y Xu L Guo L Li Y Mo M . Timing of ceiling lift–assisted out-of-bed sitting training and outcomes in critically ill patients: a prospective cohort study. Crit Care (2025) 29:493. doi: 10.1186/s13054-025-05741-9 41250213 PMC12625302

[15] Hua D Chen Y. A predictive model for 28-day mortality after discharge in patients with sepsis associated with cerebrovascular disease. Technol Health Care. (2025) 33:463-472. doi: 10.3233/THC-241150 39177630

[16] Zhao L Yang J Zhou C Wang Y Liu T. A novel prognostic model for predicting the mortality risk of patients with sepsis-related acute respiratory failure: a cohort study using the MIMIC-IV database. Curr Med Res Opin. (2022) 38:629-636. doi: 10.1080/03007995.2022.2038490 35125039

[17] Lei S Li X Zhao H Feng Z Chun L Xie Y . Risk of dementia or cognitive impairment in sepsis survivals: a systematic review and meta-analysis. Front Aging Neurosci. (2022) 14:839472. doi: 10.3389/fnagi.2022.839472 35356300 PMC8959917

[18] Hu W Shang K Chen L Wang X Li X. Comparison and combined use of NEWS2 and GCS scores in predicting mortality in stroke and traumatic brain injury: a multicenter retrospective study. Front Neurol. (2024) 15:1435809. doi: 10.3389/fneur.2024.1435809 39165267 PMC11333856

[19] De Matteis G Burzo ML Della Polla DA Serra A Russo A Landi F . Outcomes and predictors of in-hospital mortality among older patients with dementia. J Clin Med (2023) 12:59. doi: 10.3390/jcm12010059 36614856 PMC9821230

[20] Lan Y Zhu J Pu P Ni W Yang Q Chen L. Association of dementia with the 28-day mortality of sepsis: an observational and Mendelian randomization study. Front Aging Neurosci. (2024) 16:1417540. doi: 10.3389/fnagi.2024.1417540 39606027 PMC11599188

[21] Kao LT Sheu JJ Lin HC Tsai M-C Chung S-D. Association between sepsis and dementia. J Clin Neurosci. (2015) 22:1430-3. doi: 10.1016/j.jocn.2015.02.035 26165470

[22] Shen HN Lu CL Li CY. Dementia increases the risks of acute organ dysfunction, severe sepsis and mortality in hospitalized older patients: a national population-based study. PLoS ONE. (2012) 7:e42751. doi: 10.1371/journal.pone.0042751 22905169 PMC3414444

[23] Gao L Wang GD Yang XY Tong SJ Wang XJ Chen YR . Development of a risk prediction model for sepsis-related delirium based on multiple machine learning approaches and an online calculator. PLoS ONE. (2025) 20:e0323831. doi: 10.1371/journal.pone.0323831 40668815 PMC12266397

[24] Shankar-Hari M Rubenfeld GD Ferrando-Vivas P Harrison DA Rowan K. Risk factors at index hospitalization associated with longer-term mortality in adult sepsis survivors. Jama Netw Open. (2020) 3:e2013580. doi: 10.1001/jamanetworkopen.2020.13580 31150081 PMC6547123

[25] Kang C Choi S Jang EJ Joo S Jeong JH Oh S . Prevalence and outcomes of chronic comorbid conditions in patients with sepsis in Korea: a nationwide cohort study from 2011 to 2016. BMC Infect Dis. (2024) 24:184. doi: 10.1186/s12879-024-09081-x 38347513 PMC10860243

[26] Gong C Jiang Y Tang Y Liu F Shi Y Zhou H . [Elevated serum lactic acid level is an independent risk factor for the incidence and mortality of sepsis-associated acute kidney injury]. Zhonghua Wei Zhong Bing Ji Jiu Yi Xue. (2022) 34 (7):714-720. doi: 10.3760/cma.j.cn121430-20210823-01238 36100409

[27] Wang L Ma X Zhou G Gao S Pan W Chen J . SOFA in sepsis: with or without GCS. EUR J MED RES. (2024) 29:296. doi: 10.1186/s40001-024-01849-w 38790024 PMC11127461

