# Supplementary material for: Development and external validation of a 90-day mortality prediction model for comatose sepsis patients: impact of cerebrovascular disease and dementia
Source: Front Med (Lausanne). 2026 Jul 2;13:1844893. doi: 10.3389/fmed.2026.1844893 (PMC13372963; doi:10.3389/fmed.2026.1844893)
Supplement: Supplementary file 1 [file Supplementary_file_1.docx]

**Supplementary Table 1**. Candidate predictor variables and missing data handling

| Variable | Type | Missingness (%) | Handling | Retained in final model |
| --- | --- | --- | --- | --- |
| Age | Continuous | 0.0 | Complete | Yes |
| Sex | Binary | 0.0 | Complete | No (shrunk to zero) |
| Ethnicity | Categorical | 0.0 | Complete | No (shrunk to zero) |
| BMI | Continuous | 45.2 | Excluded (>40%) | — |
| SOFA score | Continuous | 3.8 | Imputed (MICE) | No (shrunk to zero) |
| GCS score | Continuous | 0.0 | Complete | Yes (retained, non-significant) |
| Mechanical ventilation | Binary | 0.0 | Complete | No (shrunk to zero) |
| Base excess | Continuous | 12.5 | Imputed (MICE) | Yes |
| PaO₂ | Continuous | 18.3 | Imputed (MICE) | Yes |
| PaCO₂ | Continuous | 52.7 | Excluded (>40%) | — |
| Lactate | Continuous | 22.4 | Imputed (MICE) | No (shrunk to zero) |
| Hemoglobin | Continuous | 48.6 | Excluded (>40%) | — |
| Creatinine | Continuous | 46.1 | Excluded (>40%) | — |
| BUN | Continuous | 8.7 | Imputed (MICE) | Yes |
| INR | Continuous | 19.2 | Imputed (MICE) | Yes |
| Congestive heart failure | Binary | 0.0 | Complete | No (shrunk to zero) |
| Cerebrovascular disease | Binary | 0.0 | Complete | Yes |
| Dementia | Binary | 0.0 | Complete | Yes |
| Renal disease | Binary | 0.0 | Complete | No (shrunk to zero) |

Note: MICE = multiple imputation by chained equations. Variables with >40% missingness were excluded from LASSO. Remaining variables with missing data were imputed using MICE with 5 imputed datasets (predictive mean matching for continuous variables, logistic regression for binary variables).

**Supplementary Table 2**. Final logistic regression model for 90-day mortality prediction in comatose sepsis patients

| Predictor | Coefficient (β) | Variable coding / Unit |
| --- | --- | --- |
| Intercept | −3.842 | — |
| Age | 0.053 | Per 1 year |
| Base excess | −0.162 | Per 1 mmol/L |
| PaO₂ | 0.042 | Per 10 mmHg decrease |
| BUN | 0.018 | Per 1 mg/dL |
| INR | 1.303 | Per 1 unit |
| Cerebrovascular disease | 0.955 | 1 = Yes, 0 = No |
| Dementia | 1.535 | 1 = Yes, 0 = No |

**Prediction formula:**

LP = −3.842 + (0.053 × Age) − (0.162 × Base excess) + (0.042 × PaO₂_decrease_per_10) + (0.018 × BUN) + (1.303 × INR) + (0.955 × Cerebrovascular disease) + (1.535 × Dementia)

P(90-day mortality) = 1 / (1 + e^(−LP))

**Worked example:** A 75-year-old patient with base excess −2 mmol/L, PaO₂ 200 mmHg (13.5 per-10-unit decrease), BUN 30 mg/dL, INR 1.5, no cerebrovascular disease, no dementia:

LP = −3.842 + 3.975 + 0.324 + 0.567 + 0.540 + 1.955 = 3.519

P = 1 / (1 + e^(−3.519)) = 97.1%

**Supplementary Table 3**. Comparison of baseline characteristics between development and external validation cohorts

| Characteristic | MIMIC-IV (n = 841) | Chinese ICU (n = 251) | P-value |
| --- | --- | --- | --- |
| Demographics |  |  |  |
| Age, median (IQR) | 70.3 (59.7–79.5) | 71.3 (60.5–80.1) | 0.42 |
| Male, n (%) | 452 (53.7) | 138 (55.0) | 0.71 |
| Physiological severity |  |  |  |
| SOFA score, median (IQR) | 5.0 (3.0–7.0) | 6.0 (4.0–8.0) | 0.03 |
| GCS score, median (IQR) | 6.0 (4.0–7.0) | 6.0 (4.0–7.0) | 0.89 |
| Invasive mechanical ventilation, n (%) | 612 (72.8) | 215 (85.7) | <0.001 |
| Laboratory parameters |  |  |  |
| Base excess, median (IQR) | 0.0 (−1.0–2.0) | −0.5 (−2.0–1.5) | 0.08 |
| PaO₂, median (IQR) | 265.0 (94.0–392.5) | 240.0 (88.0–380.0) | 0.12 |
| BUN, median (IQR) | 28.0 (18.0–45.0) | 30.0 (20.0–48.0) | 0.15 |
| INR, median (IQR) | 1.3 (1.2–1.5) | 1.3 (1.2–1.6) | 0.67 |
| Comorbidities |  |  |  |
| Cerebrovascular disease, n (%) | 152 (18.1) | 31 (12.4) | 0.04 |
| Dementia, n (%) | 71 (8.4) | 18 (7.2) | 0.58 |
| Congestive heart failure, n (%) | 198 (23.5) | 52 (20.7) | 0.41 |
| Renal disease, n (%) | 124 (14.7) | 38 (15.1) | 0.89 |
| Outcome |  |  |  |
| 90-day mortality, n (%) | 192 (22.8) | 61 (24.3) | 0.62 |

Note: Continuous variables compared using Mann-Whitney U test; categorical variables using χ² test.

**Supplementary Table 4.** Sensitivity analysis using PaO₂/FiO₂ ratio

| Variable | Coefficient (β) | OR (95% CI) | P-value |
| --- | --- | --- | --- |
| Age (per year) | 0.052 | 1.05 (1.04–1.07) | <0.001 |
| Base excess (per unit) | −0.160 | 0.85 (0.81–0.89) | <0.001 |
| PaO₂/FiO₂ (per 10-unit decrease) | 0.038 | 1.04 (1.02–1.06) | <0.001 |
| BUN (per unit) | 0.018 | 1.02 (1.00–1.04) | 0.025 |
| INR (per unit) | 1.298 | 3.66 (2.08–6.44) | <0.001 |
| Cerebrovascular disease | 0.952 | 2.59 (1.24–5.40) | 0.011 |
| Dementia | 1.532 | 4.62 (1.23–17.28) | 0.023 |
| GCS (per 1-point decrease) | 0.088 | 1.09 (0.97–1.22) | 0.128 |

Model performance: Optimism-corrected C-index 0.80 (95% CI: 0.77–0.83); AUC 0.81 (95% CI: 0.77–0.85); Brier score 0.158.

Note: PaO₂/FiO₂ ratio was calculated using the simultaneous FiO₂ recorded at the time of the worst PaO₂ measurement. Model specification otherwise identical to primary analysis.

**Supplementary Checklist 1**. Completed TRIPOD Checklist

| Section | Item | Checklist Item | Reported in |
| --- | --- | --- | --- |
| Title | 1 | Identify as development/validation of multivariable prediction model | Title, p.1 |
| Abstract | 2 | Summary of objectives, design, setting, participants, predictors, outcome, analysis, performance, conclusions | Abstract, p.1 |
| Introduction | 3a | Medical context and rationale | Introduction, p.2 |
| Introduction | 3b | Objectives, intended users, clinical utility | Introduction, p.2 |
| Methods | 4a | Study design or data source | Methods 2.1, p.3 |
| Methods | 4b | Key study dates | Methods 2.1, p.3 |
| Methods | 5a | Eligibility criteria | Methods 2.2, p.3 |
| Methods | 6a | Outcome definition and time horizon | Methods 2.3, p.3 |
| Methods | 6b | Outcome assessors and blinding | Methods 2.3, p.3 |
| Methods | 7a | All predictors defined | Methods 2.3, p.3 |
| Methods | 7b | Predictor measurement methods | Methods 2.3, p.3 |
| Methods | 7c | Predictor categories/continuous handling | Methods 2.5, p.4 |
| Methods | 7d | Blinding of predictor assessors | Methods 2.3, p.3 |
| Methods | 8 | Sample size and missing data | Methods 2.4–2.5, p.4 |
| Methods | 9a | Model development strategy | Methods 2.5, p.4 |
| Methods | 9b | Model type and building procedures | Methods 2.5, p.4 |
| Methods | 10a | Performance measures | Methods 2.5, p.4 |
| Methods | 10b | Validation approach | Methods 2.5, p.4 |
| Methods | 10c | Internal validation | Methods 2.5, p.4 |
| Methods | 10d | External validation | Methods 2.5, p.4 |
| Results | 11 | Participant flow | Figure 1, p.5 |
| Results | 12a | Participant characteristics | Table 1, p.5 |
| Results | 12b | Univariable associations | Results 3.1, p.5 |
| Results | 13a | Final model specification | Supplementary Table S1 |
| Results | 13b | Model performance | Results 3.3, Table 2, p.6 |
| Results | 13c | Model validation | Results 3.4, p.6 |
| Results | 14a | Validation performance | Results 3.4, Table 2, p.6 |
| Results | 14b | Validation calibration | Results 3.4, p.6 |
| Discussion | 16a | Limitations and generalizability | Discussion, p.8 |
| Discussion | 16b | Implications for practice | Discussion, p.8 |
| Other | 17 | Supplementary information | Supplementary Tables S1–S3, Checklist S1 |
| Other | 18 | Funding and conflicts | p.10 |


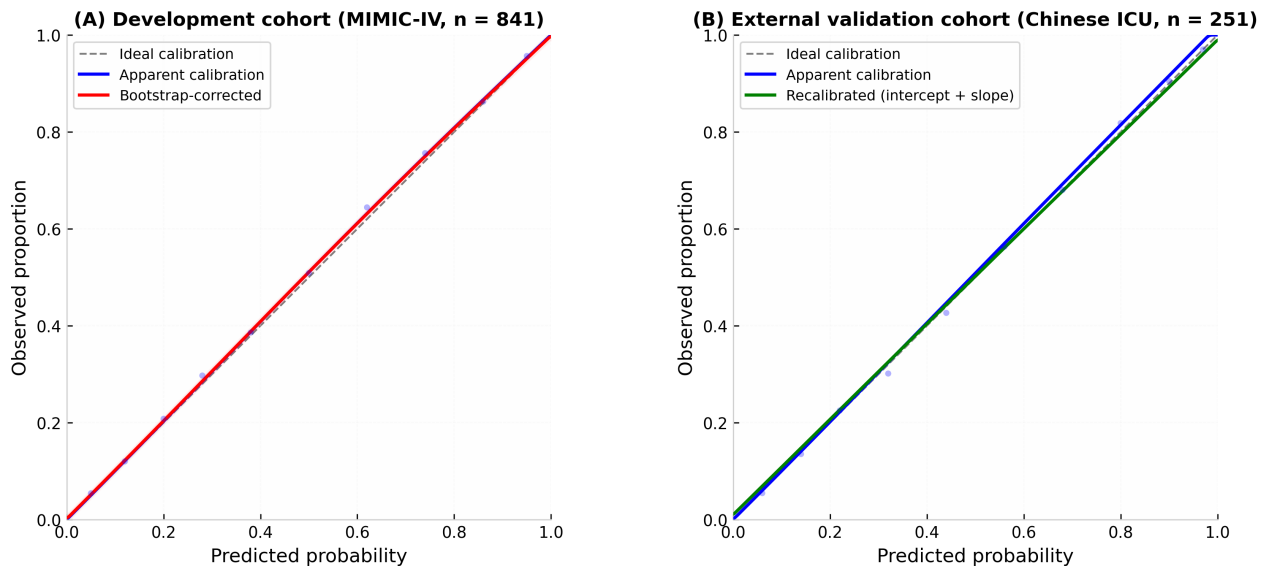


**Supplementary Figure 1.** Calibration plots for the 90-day mortality prediction model

(A) Development cohort (MIMIC-IV, n = 841). The apparent calibration curve (solid blue line) closely follows the ideal 45-degree reference line (dashed gray), with the bootstrap-corrected curve (solid red line, based on 1000 resamples) demonstrating minimal optimism.

(B) External validation cohort (Chinese ICU, n = 251). The apparent calibration curve (solid blue line) shows mild under-prediction at higher predicted probabilities. The recalibrated curve (solid green line, intercept and slope adjustment) demonstrates improved calibration.


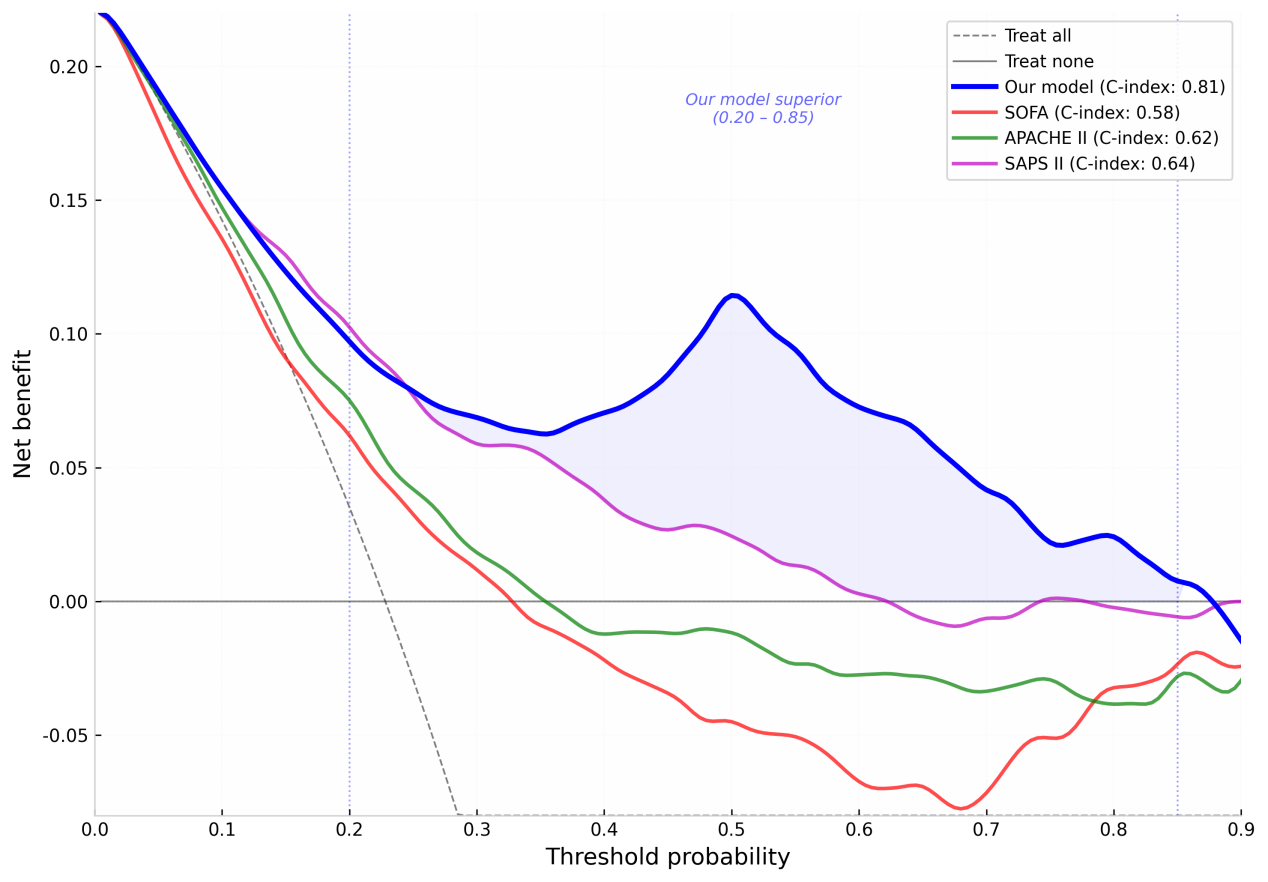
**Supplementary Figure 2.** Decision curve analysis comparing the net benefit of our 90-day mortality prediction model versus SOFA, APACHE II, and SAPS II across threshold probabilities. Our model demonstrates superior clinical utility in the threshold range of 0.20–0.85 (highlighted region).
